# Parthenolide and DMAPT exert cytotoxic effects on breast cancer stem-like cells by inducing oxidative stress, mitochondrial dysfunction and necrosis

**DOI:** 10.1038/cddis.2016.94

**Published:** 2016-04-14

**Authors:** D Carlisi, G Buttitta, R Di Fiore, C Scerri, R Drago-Ferrante, R Vento, G Tesoriere

**Affiliations:** 1 Laboratory of Biochemistry, Department of Experimental Biomedicine and Clinical Neurosciences (BioNec), University of Palermo, Polyclinic, Palermo, Italy; 2 Laboratory of Biochemistry, Department of Biological, Chemical and Pharmaceutical Sciences and Technologies (STEBICEF), University of Palermo, Polyclinic, Palermo, Italy; 3 Department of Physiology and Biochemistry, University of Malta, Msida, Malta; 4 Sbarro Institute for Cancer Research and Molecular Medicine, Center for Biotechnology, Temple University, Philadelphia, PA, USA

## Abstract

Triple-negative breast cancers (TNBCs) are aggressive forms of breast carcinoma associated with a high rate of recidivism. In this paper, we report the production of mammospheres from three lines of TNBC cells and demonstrate that both parthenolide (PN) and its soluble analog dimethylaminoparthenolide (DMAPT) suppressed this production and induced cytotoxic effects in breast cancer stem-like cells, derived from dissociation of mammospheres. In particular, the drugs exerted a remarkable inhibitory effect on viability of stem-like cells. Such an effect was suppressed by *N*-acetylcysteine, suggesting a role of reactive oxygen species (ROS) generation in the cytotoxic effect. Instead z-VAD, a general inhibitor of caspase activity, was ineffective. Analysis of ROS generation, performed using fluorescent probes, showed that both the drugs stimulated in the first hours of treatment a very high production of hydrogen peroxide. This event was, at least in part, a consequence of activation of NADPH oxidases (NOXs), as it was reduced by apocynin and diphenylene iodinium, two inhibitors of NOXs. Moreover, both the drugs caused downregulation of Nrf2 (nuclear factor erythroid 2-related factor 2), which is a critical regulator of the intracellular antioxidant response. Prolonging the treatment with PN or DMAPT we observed between 12 and 24 h that the levels of both superoxide anion and hROS increased in concomitance with the downregulation of manganese superoxide dismutase and catalase. In addition, during this phase dissipation of mitochondrial membrane potential occurred together with necrosis of stem-like cells. Finally, our results suggested that the effect on ROS generation found in the first hours of treatment was, in part, responsible for the cytotoxic events observed in the successive phase. In conclusion, PN and DMAPT markedly inhibited viability of stem-like cells derived from three lines of TNBCs by inducing ROS generation, mitochondrial dysfunction and cell necrosis.

Parthenolide (PN),^1, 2, 3^ a sesquiterpene lactone, found in medicinal plants, particularly in feverfew (*Tanacetum parthenium*), exerts selective toxicity against a wide range of tumors, ^4, 5, 6^ but is ineffective in normal cells.^7^

Structure of PN exhibits the typical *α*-methylene-*γ*-lactone group, which reacts with cysteine thiol groups inducing modifications of many biological functions. The antitumour activity of PN can be mediated by distinct mechanisms: (i) inhibition of NF-*κ*B activity either by inhibition of I*κ*B phosphorylation^8^ or alkylation of SH groups in the p65 subunit;^9^ (ii) epigenetic mechanism through DNA hypomethylation, determined by downregulation of DNA methyltransferase 1;^10^ (iii) increment in reactive oxygen species (ROS) generation. In prostate cancer cells,^11^ ROS generation is accompanied by downregulation of antioxidant enzymes, such as manganese superoxide dismutase (MnSOD) and catalase.^12^

Triple-negative breast cancers (TNBCs) are aggressive forms, associated with poor prognosis,^13^ which do not express estrogen, progesterone and Her-2/neu receptors,^13^ and consequently are unresponsive to hormonal treatment. Recently, we showed that PN and dimethylaminoparthenolide (DMAPT), a soluble analog of PN, induced cytotoxic effects in MDA-MB231 cells,^14^ the most studied line of TNBCs, by stimulating ROS generation and autophagy. Moreover, DMAPT decreased tumor growth in mice bearing xenografts of MDA-MB231 cells and induced animal survival.^14^ Finally, suberoylanilide hydroxamic acid, an histone deacetylase inhibitor, synergistically sensitized MDA-MB231 cells to PN effect.^15^

Today a body of evidence strongly suggests that a small proportion of tumor cells, termed cancer stem cells (CSCs), represent the basis for tumor formation, progression, metastasis and recurrence.^16, 17, 18^ CSCs have been demonstrated in many tumors including breast cancer.^19^

Interestingly, many drugs, which inhibit replication of the bulk of cancer cells, are not effective for eradication of CSCs in many types of tumors.^20, 21, 22^ Consequently, new researches are needed to individuate molecules able to eliminate CSCs.

This paper deals with the production of mammospheres from three distinct lines of TNBCs. Besides, it shows that both PN and DMAPT suppress production of spheres and cause cytotoxic effects in stem-like cells derived from their dissociation.

## Results

### PN inhibited production and growth of mammospheres

**Figure 1:**
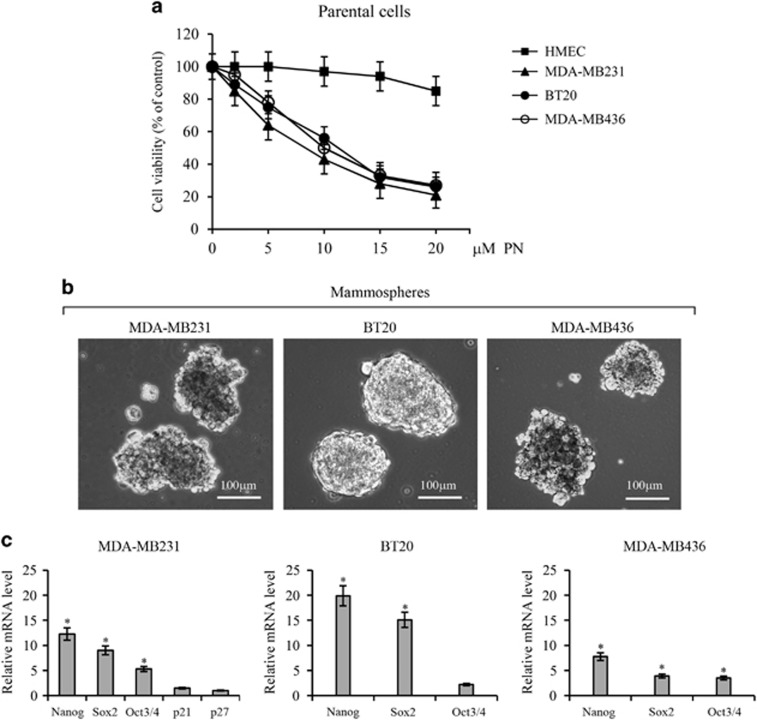
(**a**) PN inhibits viability of three different lines of TNBC cells. Dose-dependent effect evaluated at 24 h of treatment of MDA-MB231, BT20 and MDA-MB436 cells with various doses of PN in comparison with HMECs. (**b**) Images of secondary mammospheres derived from the three lines of TNBC cells under light microscopy at x200 original magnification. (**c**) Quantitative polymerase chain reaction showing the relative amounts of Nanog, Sox2, Oct3/4, p21 and p27 mRNA in stem-like cells from the three lines. Values reported in the figure express the fold difference in comparison with mRNA measured in the respective parental counterpart. In (**b**), the results are representative of three independent experiments. Scale bar, 100 *μ*m. In (**a** and **c**), the results are the mean of three independent experiments±S.D. *P<0.01 versus untreated control

As Figure 1a shows, PN inhibited viability of three distinct lines of TNBCs, namely MDA-MB231, BT20 and MDA-MB436 cells. At 5 *μ*M, PN reduced viability of the three lines by 25–40% and at 20 *μ*M by 75–80%. Otherwise, PN at 5 *μ*M was ineffective on viability of normal human mammary epithelial cells (HMECs), while at 20 μM exerted only a modest effect (−15%).

When single cells of the three lines of TNBCs were grown for 10 days in non-adherent conditions, a small number of survival cells generated floating mammospheres, as reported by other authors.^23, 24, 25^ Primary mammospheres with at least one diameter ⩾100 *μ*m were counted. MDA-MB231 cells were the most active and produced loose and not round mammospheres in the ratio of 5.4/1000 cells. Similar spheres derived from MDA-MB436 cells (1.0/1000 cells), while BT20 cells produced spheres (3.8/1000 cells) with a round conformation. Figure 1b shows typical spheres produced from the three lines of cells.

Stem cells are characterized by the expression of some genes required to maintain stem-like conditions. This group includes Nanog, Oct3/4 and Sox2. Figure 1c reports relative mRNA levels ascertained by means of RT-PCR procedure in stem-like cells derived from dissociation of secondary mammospheres produced from the three lines. Nanog exhibited a sharp increase in comparison with parental cells. Also, the levels of Oct3/4 and Sox2 increased, but less than Nanog, whereas p21 and p27, two oncosuppressor genes, showed only little modifications.

**Figure 2:**
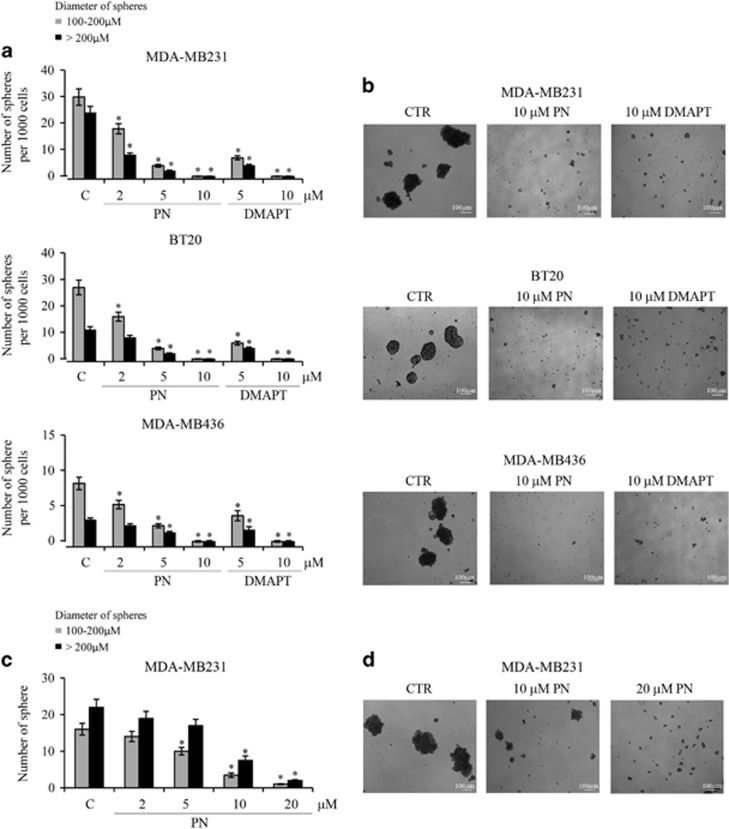
PN and DMAPT inhibited production and stability of mammospheres. (**a**) PN and DMAPT decreased in a dose-dependent manner the production of secondary mammospheres from the three lines of TNBC cells. Primary mammospheres were dissociated and the isolated cells were again grown for 10 days in non-adherent conditions, as reported in Materials and Methods, without or with PN or DMAPT at various doses. Mammospheres, with at least one diameter ⩾100 *μ*m were counted under light microscopy at x100 original magnification. (**b**) Images showing the effects of 10 *μ*M PN and 10 *μ*M DMAPT on the production of secondary mammospheres. (**c**) PN destroyed secondary mammospheres. About 40 secondary mammospheres derived from MDA-MB231 cells at 10 days of growth were treated without or with PN at various doses for other 5 days in non-adherent conditions. (**d**) Images showing the destructive power exerted by PN on secondary mammospheres derived from MDA-MB231 cells. In (**a** and **c**), the results are the mean of three independent experiments±S.D. *P<0.01 versus untreated control. In (**b** and **d**), the results are representative of three independent experiments. Scale bar, 100 *μ*m

Production of tertiary mammospheres was inhibited by PN or DMAPT in a dose-dependent manner. At 10 *μ*M both the compounds suppressed the production of spheres (Figures 2a and b).

In other experiments, various doses of PN were added in non-adherent conditions in the medium containing secondary mammospheres at day 10 of production. PN reduced their number and after 5 days, spheres were almost completely destroyed. Figures 2c and d show PN effects on spheres derived from MDA-MB231 cells. Similar results were obtained with spheres derived from the other two lines (not shown).

### PN inhibited viability of stem-like cells derived from mammospheres

Stem-like cells derived from dissociation of secondary mammospheres (sphere cells) were used for all the experiments.

**Figure 3:**
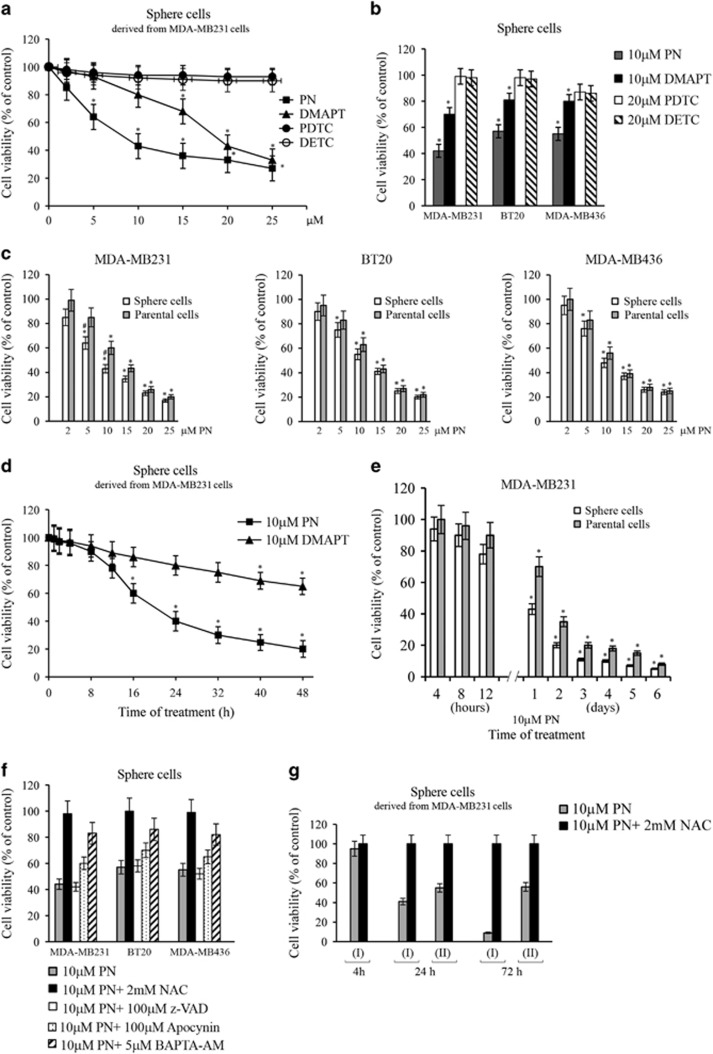
PN and DMAPT inhibited viability of mammosphere-derived cells. Secondary mammospheres, derived from three lines of TNBCs, were dissociated to produce isolated sphere cells, which were plated (8x10^3^/well) in adherent conditions. After 24 h, sphere cells were used for the experiments. At the end, viability was evaluated by MTT assay. (**a**) Dose-dependent effect evaluated at 24 h of treatment with various doses of PN or DMAPT in comparison with PDTC and DETC in MDA-MB231 sphere cells. (**b**) Comparison of the effects determined by 24 h treatment with PN, DMAPT, PDTC and DETC in sphere cells derived from the three lines of TNBCs. (**c**) Dose-dependent effect induced by 24 h of treatment with PN. Comparison between sphere cells and the respective parental cells. (**d**) Time course of the effects exerted by PN and DMAPT on MDA-MB231 sphere cells. (**e**) Comparison between the time-dependent effect exerted by 10 *μ*M PN on MDA-MB231 sphere cells and the respective parental cells. (**f**) The inhibitory influence exerted by various compounds on the effect induced by treatment for 24 h with 10 *μ*M PN in sphere cells derived from the three lines of TNBCs. (**g**) The effect of substitution of the medium in MDA-MB231 sphere cells treated with PN. (I) Control condition. Sphere cells were treated continuously with PN or PN plus NAC during the incubation. Viability was evaluated at 4, 24 and 72 h of treatment. (II) Sphere cells were treated for 4 h with PN or PN plus NAC, then the medium was substituted with fresh medium lacking in both PN and NAC and the incubation was continued. Viability was evaluated at 24 and 72 h of treatment. All the data shown in this figure are the mean of three independent experiments±S.D. *P<0.01 versus untreated control; ^#^*P<*0.01 versus parental cells

Both PN and DMAPT inhibited in a dose-dependent manner the sphere cell viability (Figures 3a and b). PN exerted a higher effect than DMAPT, in particular at low doses (2–10 *μ*M). MDA-MB231 sphere cells were the most sensible line.

PDTC (pyrrolidine dithiocarbamate) and DETC (diethyldithiocarbamate), two compounds that like PN inhibit NF-*κ*B activity, were unable to inhibit sphere cells viability, except for a modest effect on MDA-MB436 sphere cells (Figures 3a and b). Therefore, viability in stem-like cells is not correlated with NF-*κ*B activity.

MDA-MB231 sphere cells were more susceptible than parental cells to both PN (Figure 3c) and DMAPT (not shown). Significant differences were observed at 5 and 10 *μ*M. Instead, differences between sphere and parental cells were not statistically reliable for the two other cell lines.

PN and DMAPT progressively decreased viability of MDA-MB231 sphere cells so that at 24 h of treatment with 10 *μ*M PN viable cells lowered to ~40% of control (Figures 3d and f) and after 6 days (Figure 3e) to only 4%. A similar behavior was observed using BT20 and MDA-MB436 sphere cells (not shown).

As Figure 3f shows, PN inhibitory effect on cell viability was not modified by z-VAD, a general inhibitor of caspases, but was suppressed by *N*-acetylcysteine (NAC). Therefore, the effect on cell viability was not a consequence of apoptosis, but of oxidative stress. Moreover, apocynin (100 *μ*M), an inhibitor of NADPH oxidase (NOX),^26, 27^ and BAPTA-AM (1,2-bis-(o-aminophenoxy)-ethane-*N*,*N*,*N*′,*N*′-tetraacetic acid, tetraacetoxymethyl ester; 5 *μ*M), an intracellular Ca^2+^ chelator,^28^ partially prevented PN effect, since in their presence PN decreased viability to 60% and 83%, respectively.

In some experiments, MDA-MB231 sphere cells were at first treated for 4 h with 10 *μ*M PN, then the medium was substituted with fresh medium lacking in PN and the incubation was started again (condition II). In this case, at 24 h of incubation sphere cell viability decreased to 56% of control (Figure 3g). This was a remarkable effect, although minor than that observed in condition I, when PN was maintained continuously for the entire treatment. The inhibitory effect was suppressed in both the conditions by 2 mM NAC. By prolonging the incubation until 3 days, viability further decreased in condition I to a very low level, while in condition II remained at the level found at 24 h.

### PN induced ROS generation in stem-like cells

Sphere cells were treated for various times (1–24 h) with 10 *μ*M PN and the effects were analyzed using three different fluorescent probes, such as dichlorofluorescein (DCF), dihydroethidium (DHE) and hydroxyphenyl fluorescein (HPF).

**Figure 4:**
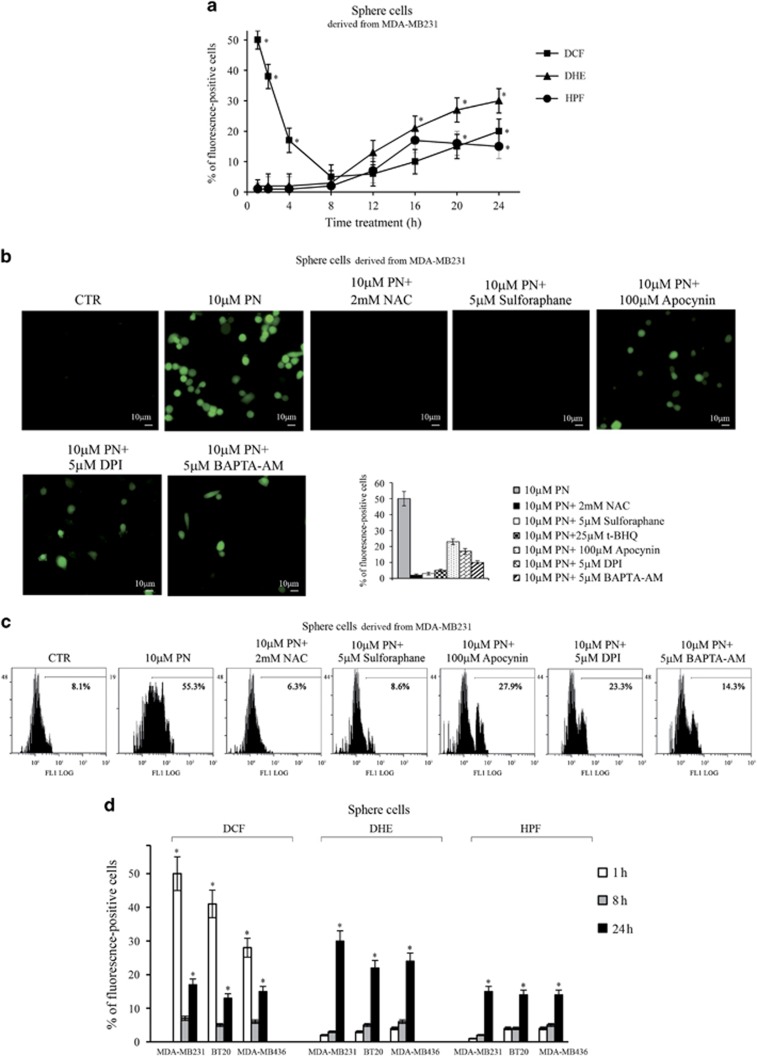
PN induced generation of oxygen radicals in sphere cells derived from three lines of TNBCs. Sphere cells (8 × 10^3^ cells per well) were treated for various times (1–24 h) with 10 *μ*M PN. Three different fluorescent probes were used: DCF, DHE and HPF. Fluorescent cells were visualized with a Leica DC 300 F microscope at x200 original magnification with fluorescent filters for FITC (DCF and HPF) or rhodamine (DHE). Cells with fluorescence were counted in three different microscopic fields in each well (3 wells per treatment) and expressed as percentage of the total number of cells counted under light microscopy. (**a**) The time course of DCF, DHE and HPF signals analyzed by fluorescence microscopy in MDA-MB231 sphere cells treated with 10 *μ*M PN. (**b**) Images of fluorescence microscopy showing the inhibitory influence exerted in MDA-MB231 sphere cells by various compounds on the effect induced on DCF signal by treatment for 1 h with 10 *μ*M PN. In the inset, histograms showing the percentage of fluorescence-positive cells observed in the various conditions. (**c**) Flow cytometric detection of DCF signal in the same conditions shown in (**b**). (**d**) The effect of 10 *μ*M PN on the amount of positive cells to DCF, DHE and HPF signals analyzed by fluorescence microscopy for various times in sphere cells derived from the three lines of TNBCs. In (**a**, **b**, inset and **d**), the results are the mean of three independent experiments±S.D. *P<0.01 versus untreated control. In (**b**, images and **c**), the results are representative of three independent experiments. Scale bar, 10 μm

Figure 4a shows the time course of the three signals analyzed in MDA-MB231 sphere cells by fluorescence microscopy. DCFH-DA is a fluorescent probe widely used for H_2_O_2_, but it also reacts strongly with hROS. Ten micromoles of PN rapidly increased the amount of cells positive to DCF signal; at 1 h of treatment, ~50% of sphere cells resulted positive to DCF, while with 10 *μ*M DMAPT only 25% were positive (not shown). Then with PN the percentage decreased until 8 h to only 6%. Afterwards, again increased, reaching at 24 h the value of 17%.

Figure 4b reports images of fluorescence microscopy showing that PN effect at 1 h of treatment was suppressed by 2 mM NAC and markedly reduced by apocynin and DPI, two inhibitors of NOX, and by BAPTA-AM. Interestingly, 5 *μ*M sulforaphane (SF)^29^ and 25 *μ*M *tert*-butylhydroquinone (tBHQ),^30^ two activators of Nrf2, suppressed PN effect on DCF signal. Histograms, reported in the inset of Figure 4b, indicate the percentages of fluorescent-positive cells estimated in the various conditions. The results were confirmed by cytofluorimetric analysis shown in Figure 4c.

Superoxide radical was detected by using the fluorochrome DHE (Figure 4a). DHE-positive cells were not observed in the first 8 h. Afterwards, their number progressively increased, reaching at 24 h the percentage of ~27% of the total cell number.

hROS were detected by using the fluorescent probe HPF (Figure 4a). Also in this case positivity to HPF, proving production of hydroxyl radicals and peroxynitrite, was observed after the first 8 h of treatment.

Finally, 10 *μ*M PN strongly induced ROS generation also in BT20 sphere cells, while a minor effect was ascertained in MDA-MB436 sphere cells (Figure 4d).

In parental cells, as observed previously,^31^ PN stimulated ROS generation but at higher concentration (15–25 *μ*M), while 10 *μ*M was ineffective.

### PN downregulated Nrf2 in sphere cells

**Figure 5:**
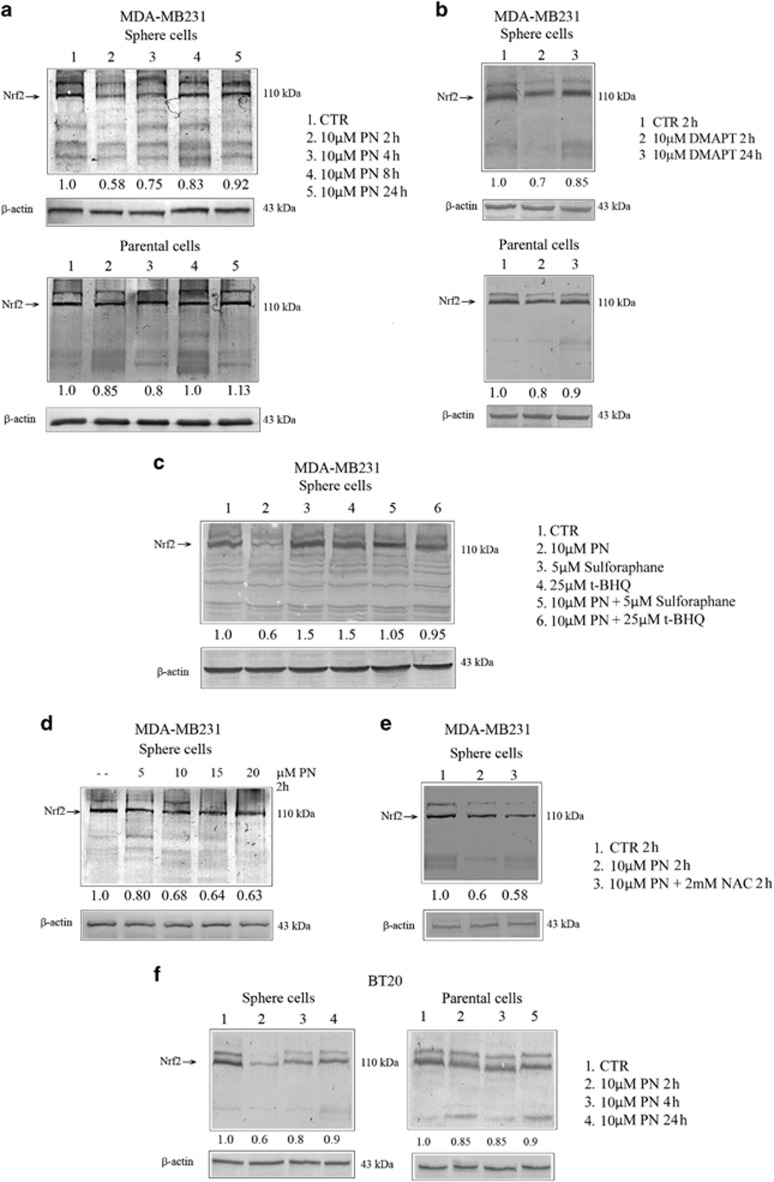
Western blotting analysis showing the effects of PN and DMAPT on the expression of Nrf2. Ten micromoles of PN (**a**) and 10 *μ*M DMAPT (**b**) caused downregulation of Nrf2 expression in MDA-MB231 sphere cells. Time dependence of the effect in comparison with the parental cells. (**c**) SF and tBHQ suppress downregulation of Nrf2 induced by PN in MDA-MB231 sphere cells. Sphere cells were previously incubated for 2 h without or with the activators of Nrf2, SF or tBHQ. Then, 10 *μ*M PN was added and the treatment was protracted for other 2 h. (**d**) The dose dependence of PN effect in MDA-MB231 sphere cells. (**e**) The addition of 2 mM NAC did not modify PN effect observed at 2 h of treatment. (**f**) The time dependence of PN effect in BT20 sphere cells in comparison with the parental cells. All the results are representative of three independent experiments

We investigated about the effect of both PN and DMAPT on the expression of Nrf2, a critical regulator of the intracellular antioxidant response.^32, 33^ Western blotting analysis demonstrated that treatment of MDA-MB231 sphere cells for 2 h with 10 *μ*M PN decreased Nrf2 expression by 40% (Figure 5a). Then, the level increased with the time of incubation, reaching at 24 h a value near to the control. A minor effect on Nrf2 expression was exerted by DMAPT (Figure 5b). PN effect was much higher in sphere cells compared with that in the parental counterpart, where PN treatment for 2 h decreased Nrf2 level by only 15%. Five micromoles of SF or 25 *μ*M tBHQ, two activators of Nrf2, increased in sphere cells the basal level of Nrf2 (Figure 5c). Furthermore, both SF and tBHQ suppressed Nrf2 downregulation induced by PN. The effect on Nrf2 was dose-dependent (Figure 5d). At 2 h of treatment, a clear decrease in the intensity of Nrf2 band was observed already with 5 *μ*M PN; then, the effect increased with PN dose, reaching the maximum with 15 *μ*M. As 2 mM NAC (Figure 5e) did not modify PN effect, downregulation of Nrf2 was not a consequence of oxidative stress.

PN decreased Nrf2 level also in BT20 sphere cells (Figure 5f) with a similar effect to that observed in MDA-MB231 sphere cells.

### PN induced in sphere cells dissipation of mitochondrial membrane potential and caused cell necrosis

As PN inhibited sphere cell viability in particular between 8 and 24 h of treatment, we investigated about the events occurring during this phase. At first we studied, using the fluorescent cationic dye JC-1 (5,5′,6,6′-tetrachloro-1,1′,3,3′-tetraethylbenzimidazolylcarbocyanine iodide), whether the exposure to PN modified mitochondrial membrane potential (Δψm).

**Figure 6:**
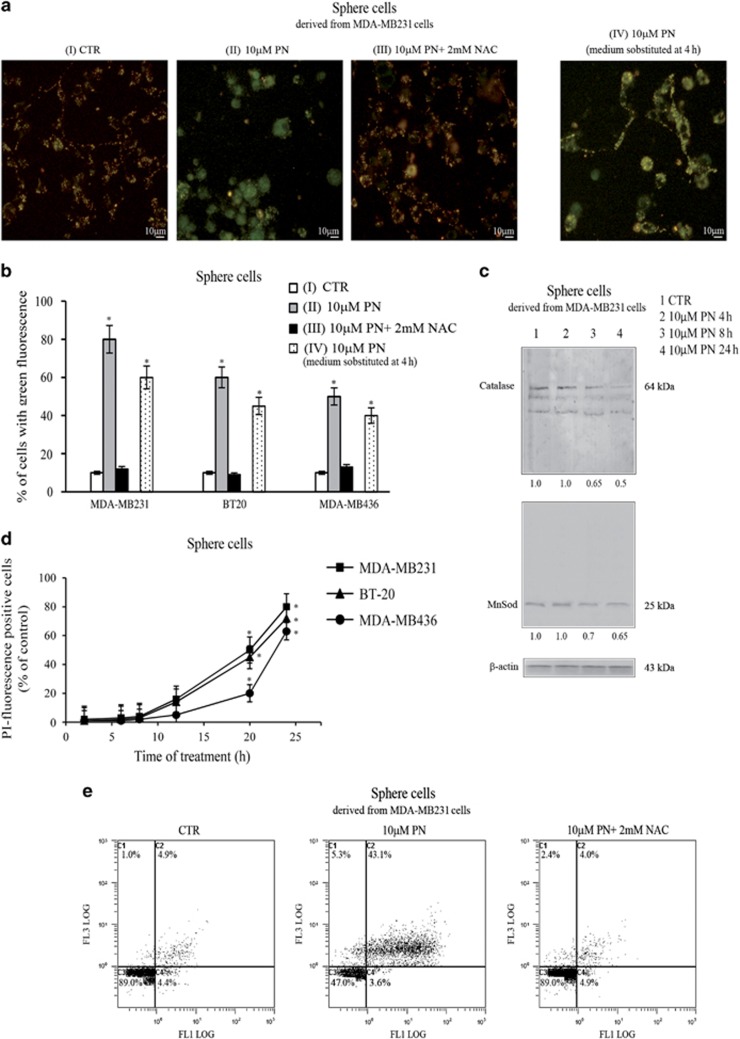
PN caused mitochondrial depolarization and necrosis of sphere cells derived from three lines of TNBCs. (**a** and **b**) Estimation of Δψm. Sphere cells (8 × 10^3^ cells per well) were treated for 24 h with 10 *μ*M PN without or with 2 mM NAC. In condition IV, cells were treated with PN for only 4 h, then the medium was substituted with fresh medium lacking in PN and the incubation was protracted until 24 h. At the end of incubation in all samples, the fluorescent cationic dye JC-1 was added for 15 min and then fluorescence was visualized by a Leica microscope at x200 original magnification with fluorescent filters for FITC and rhodamine. Fluorescent sphere cells were counted in three different microscopic fields in each well (3 wells per treatment) and expressed as percentage of the total number of cells counted under light microscopy. (**a**) Merged images of an experiment performed using MDA-MB231 sphere cells. (**b**) Histograms showing the percentage of cells with green fluorescence ascertained for the three lines of sphere cells. (**c**) Western blotting analysis showing the effect induced by treatment for various times with 10 *μ*M PN on the expression of MnSOD and catalase in MDA-MB231 sphere cells. (**d**) Time course of the effect exerted by 10 *μ*M PN on the number of PI-positive cells. Sphere cells from the three lines of TNBCs were treated with 10 *μ*M PN for various times, then PI was added as reported in Materials and Methods and the percentages of PI-positive cells were ascertained. (**e**) Analysis by Annexin V/PI double-staining assay. After 24 h of treatment with 10 *μ*M PN, without or with 2 mM NAC, MDA-MB231 sphere cells were stained with Annexin V-FITC and PI and analyzed by flow cytometry. In (**a**, **c** and **e**), the results are representative of three independent experiments. Scale bar, 10 *μ*m. In (**b** and **d**), the results are the mean of three independent experiments±S.D. *P<0.01 versus untreated control

When MDA-MB231 sphere cells were incubated without PN, red-orange fluorescence prevailed on greenish fluorescence, suggesting that most of the cells were polarized. Greenish fluorescence markedly increased after treatment with 10 *μ*M PN, indicating dissipation of Δψm. The effect already appeared at 12 h and reached the maximum at 24 h, when most of the cells were depolarized. Two micromoles of NAC suppressed Δψm dissipation induced by PN. Figure 6a (conditions I, II and III) shows merged images relative to sphere cells, treated for 24 h with PN. In condition IV, the medium containing PN was substituted after 4 h of treatment with fresh medium lacking in PN and the incubation was continued. Also in this case greenish fluorescence clearly prevailed in merged images at 24 h of incubation, but the efficacy of the treatment was minor. PN induced dissipation of Δψm also in sphere cells derived from the two other lines of TNBCs (Figure 6b), but with a lower efficacy in comparison with MDA-MB231 sphere cells.

In agreement with previous observations reported for prostate cancer cells,^12^ 10 *μ*M PN decreased in MDA-MB231 sphere cells both MnSOD and catalase levels (Figure 6c). The effects appeared at 8 h of treatment and reached the maximum at 24 h, in concomitance with the increment in the levels of superoxide anion and hROS.

To study the time course of the cytotoxic effect induced by PN, sphere cells were treated with propidium iodide (PI). The amount of PI-positive cells increased very slowly in the first 8 h of treatment with 10 *μ*M PN, then more rapidly, in accordance with PN effect exerted on cell viability, reaching for MDA-MB231 sphere cells at 24 h the proportion of 80% of the total cells (Figure 6d), while for BT20 and MDA-MB436 sphere cells, PI-positive cells reached 70% and 65%, respectively.

Interestingly, when the medium containing PN was substituted after 4 h of treatment with medium lacking in PN, changes in MnSOD and catalase levels were not observed in MDA-MB231 sphere cells at 24 h of treatment. Moreover, in this case the amount of PI-positive sphere cells reached at 24 h only the proportion of 33% (not shown).

To ascertain the mechanism of cytotoxic effect, sphere cells were stained with Annexin V-FITC (fluorescein isothiocyanate)/PI and analyzed by flow cytometry at various times of treatment with 10 *μ*M PN. Using MDA-MB231 sphere cells, population of positive cells to both Annexin V and PI (C2) increased during treatment, reaching at 24 h the percentage of 43.1% (Figure 6e). Although these cells could be either late apoptotic or necrotic dead cells, we concluded that they are necrotic dead cells, as modest amounts of early apoptotic cells (C4) were found during the treatment, and at 24 h cells in C4 accounted for 3.6%, a value inferior than that found in the control (4.4%). Instead, cells undergoing necrosis (C1) exhibited a different trend and at 24 h their amount increased from 1.0% of control to 5.3% of treated cells. PN effect was completely abolished by 2 mM NAC. Similar results were obtained using BT20 and MDA-MB436 sphere cells. In particular, cells in C2 reached at 24 h of treatment the percentages of 40.2% and 38.6%, respectively.

## Discussion

Regenerative ability of tumors depends on a small population of self-renewing CSCs.^17, 34^ Proliferation of normal stem cells is submitted to a control leading to the production of various typologies of cells. CSCs are lacking in this control and in the active state induce a progressive increase in undifferentiated cells causing the generation of tumors. Besides, CSCs exhibit many protective systems,^35^ consisting in multifunctional efflux transporters and in mechanisms directed against apoptosis. Therefore, CSCs display clinical resistance to chemotherapeutic agents and to radiation and after conventional treatment can remain vital, although in a silent state. However, CSCs can be activated by the surrounding microenvironment,^36, 37^ leading to recurrence or distant metastasis. Consequently, new approaches to kill CSCs and to eliminate cancer recurrence represent a new possible strategy against tumors.

PN was identified in leukemia and in solid tumors as the first small molecule capable of killing CSCs.^38^ Recently, Zhou *et al.*^24^ showed that PN inhibited the production of mammospheres from breast cancer MCF-7 cells, an effect that was caused by inhibition of NF-*κ*B activity.

TNBCs often exhibit acquired resistance when submitted to standard chemotherapy^39^ and undergo recurrence and metastasis. As these events depend on stem cell activity, we investigated in this research about the effects exerted by PN and DMAPT on stem-like cells. We ascertained that both PN and DMAPT suppressed the production of mammospheres from the three lines of cells. Besides, the two compounds inhibited viability of stem-like cells prepared from mammospheres. This inhibitory effect was clearly evident for MDA-MB231 sphere cells at low doses (2–10 *μ*M) of PN.

To clarify the mechanism of these effects, we investigated about the role of PN in ROS generation, as suggested by two considerations: (i) intracellular ROS level, which is essential in cell viability control, results from the balance between the mechanisms that produce ROS and the antioxidant activities. In cancer cells, discrete amounts of ROS are involved in the stimulation of prosurvival pathways,^40, 41^ but increment in ROS level causes structural damages and activation of antisurvival pathways. (ii) Previously, we showed^14^ that the cytotoxic effect exerted by PN in MDA-MB231 cells is correlated with ROS generation.

Interestingly, ROS level in breast CSCs is lower compared with that in non-stem counterparts.^42, 43^ Moreover, it seems that the intracellular amount of ROS must remain at low levels to assure stem cell viability. Consequently, the increase in ROS level could represent an effective mechanism for the induction of CSC death.

Our results show that DCF-positive sphere cells markedly enhanced at 1 h of PN treatment with PN. DCF signal is induced not only by H_2_O_2_ but also by hROS. As the specific probe HPF showed that in the first hours of treatment hROS were not detected, it seems that the positivity to DCF signal was entirely due to H_2_O_2_ production. Furthermore, as DCF-positive cells were markedly decreased by both apocynin and DPI, two effective inhibitors of various forms of NOXs, we suggest that NOXs have a role in ROS generation in the first hours of treatment, although other mechanisms can be involved in this process. Finally, also BAPTA-AM markedly reduced positivity to DCF signal. Therefore, PN could induce NOX activation through a Ca^2+^-dependent mechanism. In this regard, it was demonstrated that Ca^2+^ regulates activation and translocation of Rac,^44^ a component of NOX complex, and that elevation of intracellular Ca^2+^ stimulates NOX5 to generate superoxide.^45^

To ascertain whether the increase in H_2_O_2_ level can also result from a decrease in the antioxidant activities, we investigated about the effect of PN on Nrf2 expression. The transcription factor Nrf2 controls the expression of many antioxidant and detoxifying genes.^32, 33^ Nrf2 is regulated by Keap1,^46, 47, 48^ a factor that mediates ubiquitination and the consequent proteasomal degradation of Nrf2. Recently, it has been shown that brusatol, an inhibitor of Nrf2 pathway, markedly decreases the Nrf2 level,^49, 50^ while it enhances intracellular ROS and sensitizes tumor cells to chemotherapeutic drugs. These results demonstrate that Nrf2 exerts an important role in the control of the antioxidant response.

This paper shows that PN downregulates Nrf2 expression in sphere cells, an effect suppressed by both SF and tBHQ, two activators of Nrf2. Downregulation of Nrf2, which was observed after a brief period of treatment with PN, could be, at least partially, responsible for the rapid induction of ROS level caused by PN. This conclusion is supported by the observation that both SF and tBHQ markedly reduced PN effect on DCF signal.

In conclusion, we show that 10 *μ*M PN and DMAPT increased in stem-like cells the H_2_O_2_ level in the first hours of treatment, whereas at this dose they were ineffective on the parental counterparts. Although it seems clear that NOX activation and Nrf2 downregulation are implicated in PN activity, however, the exact mechanisms by which the drugs increased ROS level in stem-like cells are unclear at the moment. Therefore, these aspects deserve new investigations. With regard to PN effect on the Nrf2 level, we hypothesize that PN can act similarly to brusatol,^51^ which reduces the Nrf2 level through enhanced ubiquitination and degradation of Nrf2 protein.

In the second phase of treatment (12–24 h), dysfunction of mitochondrial activity occurred, as suggested by dissipation of Δψm, which increased from 12 h to 24 h, when most of the cells were depolarized. In this regard, the role of ROS is suggested by the suppression of mitochondrial depolarization determined by NAC. Mitochondrial dysfunction can depend on intracellular thiol depletion, caused by ROS generation in the first hours of treatment. Moreover, in the second phase of treatment mitochondrial dysfunction, together with the decrement in MnSOD and catalase levels, can indeed stimulate the production of radical species (superoxide anion and hROS), and these can further increase the mitochondrial dysfunction and cytotoxic effects. A relationship between ROS generation and thiol depletion was previously demonstrated by us in TNBC cells.^14^

During the second phase of treatment in concomitance with mitochondrial depolarization, viability of sphere cells exhibited a progressive decrement. Accordingly, the amount of PI-positive cells progressively increased. However, PN and DMAPT did not stimulate apoptotic events, as their effect on cell viability was not prevented by z-VAD. Moreover, Annexin V/PI test showed that the drugs caused a progressive increment in necrotic cells between 12 and 24 h. In conclusion, PN and DMAPT also at low doses induced the generation of radical species and mitochondrial depolarization with the consequent cell necrosis.

It seems that H_2_O_2_ generated in the first hours of treatment was partially responsible for the cytotoxicity observed in the successive phase. In fact, when the cells were treated with PN only for the first 4 h and then the drug was removed, a partial decrement in cell viability and a partial depolarization of sphere cells were observed. However, the persistent presence of PN also in the second phase was a decisive to induce downregulation of MnSOD and catalase, as well as to increase depolarization of sphere cells and cell necrosis.

Chemotherapy with platinum agents, anthracyclines and taxanes represents today the elective treatment for TNBCs.^38^ As this therapy is accompanied by a high rate of recidivism, a novel treatment strategy is urgently needed.^52, 53^ Our results, concerning the effects exerted by PN and DMAPT on stem-like cells derived from TNBCs, strongly suggest that the two drugs can be used for new therapeutic strategies against TNBCs. Moreover, association with other drugs could be useful to increase oxidative stress and cytotoxic effect induced by PN. Finally, it seems possible that PN, increasing ROS level, can sensitize breast CSCs to radiation, as previously ascertained in prostate cancer cells.^12, 54^ This consideration suggests a combination of PN treatment with irradiation to improve the effectiveness of therapy for TNBCs.

## Materials and Methods

### Chemicals and reagents

PN was supplied by Sigma-Aldrich (Milan, Italy), whereas DMAPT was supplied by Biomol (Plymouth Meeting, PA, USA). All the other reagents were purchased from Sigma-Aldrich, except for z-Vad-fmk, which was supplied by Promega (Milan, Italy).

### Cell cultures

HMECs were purchased from Lonza (Walkersville, MD, USA) and grown according to the manufacturer's instructions. Three different lines of TNBC cells, supplied by ‘Istituto Scientifico Tumori' (Genoa, Italy), were used for these experiments. MDA-MB231 cells were grown as a monolayer in Dulbecco's modified Eagle's medium (DMEM), BT20 cells in minimum essential medium (MEM) and MDA-MB436 cells in Roswell Park Memorial Institute (RPMI-1640). The three media were supplemented with 10% fetal calf serum, 2 mM glutamine, 1% non-essential amino acids and 1 mM pyruvate. Cells were grown in an incubator at 37 °C in a humidified atmosphere containing 5% CO_2_. Before each experiment, cells were seeded in 96- or 6-well plates and were allowed to adhere overnight, and then were treated with chemicals or vehicle only.

### Mammosphere culture

Single cells of MDA-MB231, BT20 and MDA-MB436 lines were plated in ultralow attachment plates (Corning Incorporated Life Sciences, Corning, NY, USA) at a density of 5 000 viable cells per ml either in primary cultures or in the successive passages and grown in DMEM/Ham's F12 (Euroclone, Milan, Italy) without bovine pituitary extract, but supplemented with B27 (Life Technologies, Eugene, OR, USA), 20 ng/ml EGF, 20 ng/ml bFGF (Life Technologies) and 5 *μ*g/ml Insulin (Sigma-Aldrich). Mammospheres were collected by gentle centrifugation after 10–12 days and dissociated enzymatically with 0.05% trypsin, 0.02% EDTA (Sigma-Aldrich) and mechanically by a glass Pasteur pipette. Dissociated cells were passed through a 40-μm sieve and analyzed microscopically for single cells and used for the various experiments. Three passages were performed at intervals of 10–12 days.

### Cell viability and cell death assays

Cell viability was ascertained, as described previously,^14, 15^ by MTT method, a colorimetric assay. For these experiments, cells (8 × 10^3^/well) were plated in 200 *μ*l of DMEM in a 96-well plate and treated for various times with PN or DMAPT or other compounds. Control samples were incubated for the established times in DMEM supplemented with vehicle only. At the end, the absorbance was measured directly at 490 nm in a 96-well plate using an automatic ELISA plate reader (OPSYS MR; Dynex Technologies, Chantilly, VA, USA).

To determine extensive membrane damage, cells were treated, as suggested by Asare *et al.*,^55^ with PI, a cell-impermeable nuclear dye, which stains the nuclei of cells that have lost plasma membrane integrity. After treatment of the cells (8 × 10^3^/well) with PN or DMAPT, PI (2.0 *μ*g/ml medium) was added and the incubation was protracted for additional 15 min. At the end, cell morphology was visualized by a Leica MDR microscope equipped with a DC300F camera (Leica, Wetzlar, Germany) using rhodamine filter to examine PI with an excitation wavelength of 596 and emission wavelength of 620 nm. Cells with red fluorescence were counted and normalized to total number of cells per field to calculate the percentage of PI-positive cells.

Apoptotic and necrotic effects were identified by using the Annexin V-FITC/PI Detection Kit (BD Biosciences, Pharmingen, San Diego, CA, USA) according to the manufacturer's instructions. Fluorescence of the cells was analyzed by flow cytometry on a Beckman Coulter Epics XL Flow Cytometer (Brea, CA, USA).

### Analysis of Δψm

The Δψm in CSCs was measured using the cationic dye JC-1, which in depolarized mitochondria shows a fluorescence shift from red to green. Consequently, dissipation of Δψm was indicated by an increase in the green-to-red fluorescence-intensity ratio. After treatment with the drugs, cells (8 × 10^3^/well) were incubated with medium containing JC-1 (Cayman Chemical Company, Ann Arbor, MI, USA) for 15 min at 37 °C and then washed two times with PBS. Then, the cells were analyzed on a Leica DMR fluorescence microscope (Leica Microsystems, Wetzlar, Germany) by using appropriate filters for rodhamine (excitation wavelength of 596 nm and emission wavelength of 620 nm) and FITC (excitation wavelength of 485 nm and emission wavelength of 530 nm).

### Evaluation of ROS generation

To ascertain the effect exerted by both PN and DMAPT on ROS generation, we analyzed by fluorescence microscopy the changes produced by the two compounds on three different fluorescent signals: DCF, DHE and HPF.

H_2_-DCFDA (5-(and-6)-carboxy-2′,7′-dichlorodihydrofluorescein diacetate; Molecular Probe, Life Technologies, Eugene, OR, USA) is a cell-permeant, fluorogenic dye that easily diffuses across cell membranes. After cleavage of acetate groups by intracellular esterases, a fluorescent adduct (DCF) is produced by oxidation. This probe is widely used for H_2_O_2_. However, the probe lacks specificity as it also reacts with hROS,^56^ such as hydroxyl radical and peroxynitrite. Cells (8 × 10^3^/well) were treated with the effectors for various times. Then, the medium was removed, 100 *μ*l of 50 *μ*M H_2_-DCFDA were added and the incubation was protracted for 30 min at 37 °C.

Production of superoxide anion was assessed by DHE (Sigma-Aldrich) staining. The fluorochrome DHE is oxidized by superoxide to fluorescent ethidium that intercalates with nuclear DNA, staining the nucleus with a bright red fluorescence. After treatment with PN, the cells were incubated with 20 *μ*M DHE in PBS for 15 min at 37 °C in darkness.

HPF (Enzo Life Sciences Inc., Farmingdale, NY, USA) was used to detect hROS (hydroxyl radical and peroxynitite). HPF is a cell-permeable minimally fluorescent dye, which reacts with hROS, and is converted to fluorescein, which exhibits strong, dose-dependent fluorescence.^56^ For this analysis, cells, after the treatment, were incubated with 10 *μ*M HPF in PBS for 1 h at 37 °C in the dark.

In all the three cases at the end of incubation, the medium containing the fluorescent signal was replaced with PBS alone and the fluorescence was directly visualized by means of a fluorescence miscroscope.

All analyses were performed by a Leica DMR fluorescence microscope equipped with a DC300F camera with appropriate filters. FITC filter with excitation wavelength of 485 nm and emission wavelength of 530 nm was used for the analysis of DCF and HPF signals, while rhodamine filter with excitation wavelength of 596 nm and emission wavelength of 620 nm was used for DHE analysis. All the images were acquired by the Leica Q Fluoro Software (Wetzlar, Germany). Cells with green or red fluorescence were counted and normalized to a total number of cells per field to calculate the percentage of cells producing ROS. Three fields per condition were analyzed.

Positivity of sphere cells to DCF signal was also evaluated by flow cytometer Epics XL (Brea, CA, USA). Cells (2x10^5^/well) were treated with the effectors for 1 h. Then, the cells were harvested and incubated with 50 *μ*M H_2_-DCFDA for 30 min at 37 °C in darkness. After centrifugation (120x*g* for 5 min), cells were resuspended in PBS and analyzed using flow cytometer with excitation and emission setting at 480 and 525 nm, respectively.

### RNA extraction and real-time PCR analysis

RNA was extracted by Trizol reagent (Life Technologies Ltd, Monza, Italy) and isolated using Direct-zol RNA MiniPrep Kit (Zymo Research, Irvine, CA, USA). Then, after removal of residual genomic DNA with DNase I (Zymo Research), oligo (dT)-primed reverse transcription was performed on 1 *μ*g of total cellular RNA using the iScript cDNA Synthesis Kit (Bio-Rad Laboratories Srl, Milan, Italy), following the manufacturer's instructions. For real-time PCR analyses, each cDNA sample was amplified by IQ SYBR Green Supermix (Bio-Rad Laboratories), as reported previously,^57^ using the QuantiTect primers Oct3/4, Sox2, Nanog^58^ (Qiagen, Milan, Italy). All PCR reactions were performed in triplicate in 96-well plates; each reaction mixture contained 2 *μ*l of template cDNA, 10 *μ*l of SYBR Green PCR Master Mix 2X (Bio-Rad Laboratories), forward and reverse primers at the concentration of 300 nM and RNase-free dH_2_O to a final volume of 20 *μ*l. Reactions were performed in iQ5 Thermal Cycler Instrument (Bio-Rad Laboratories), as reported previously.^58^ The relative quantities of analyzed genes were calculated using the 2^–ΔΔCt^ method and the data were normalized with the endogenous control, GAPDH (Qiagen).

### Western blotting analysis

Cell lysates and protein samples were prepared as reported previously.^57^ Equal amounts of protein samples (50 *μ*g per lane) were run in a SDS-polyacrylamide gel electrophoresis, and then transferred to a nitrocellulose membrane. All analyses were performed using specific primary antibodies, which were provided by Santa Cruz Biotechnology (Santa Cruz, CA, USA). Then, the detection was developed by using a secondary antibody conjugated with alkaline phosphatase. Protein bands were visualized using nitroblue tetrazolium and 5-bromo-4-chloro-3-indoyl-phosphate (Promega, Milan, Italy) and their intensity was quantified by densitometric analysis using the SMX Image software (Bio-Rad Laboratories). The correct protein loading was ascertained by red Ponceau staining and immunoblotting for *β*-actin. All the blots shown are representative of at least three different experiments.

### Statistical analysis

Results are presented as mean±S.D. of data from at least three independent experiments. Data were analyzed using Student's *t*-test. A *P-*value below 0.01 was considered significant.

## Abbreviations

BAPTA-AM: 1,2-bis-(o-aminophenoxy)-ethane-*N*,*N*,*N*',*N*-tetraacetic acid, tetraacetoxymethyl ester
BCIP: 5-bromo-4-chloro-3-indoyl-phosphate
CSC: cancer stem cell
DETC: diethyldithiocarbamate
DHE: dihydroethidium
DMAPT: dimethylaminoparthenolide
DPI: diphenylene iodinium
Δψm: mitochondrial membrane potential
FITC: fluorescein isothiocyanate
H2-DCFDA: 5-(and-6)-carboxy-2′,7′-dichlorodihydrofluorescein diacetate
HPF: hydroxyphenyl fluorescein
JC-1: 5,5′,6,6′-tetrachloro-1,1′,3,3′-tetraethylbenzimidazolylcarbocyanine iodide
MnSOD: manganese-dependent superoxide dismutase
NAC: *N*-acetylcysteine
NOX: NADPH oxidase
Nrf2: nuclear factor erythroid 2-related factor 2
PDTC: pyrrolidine dithiocarbamate
PI: propidium iodide
PN: parthenolide
ROS: reactive oxygen species
SAHA: suberoylanilide hydroxamic acid
SF: sulforaphane
tBHQ: *tert*-butylhydroquinone
TNBC: triple-negative breast cancer
